# MRI imaging features of HIV-related central nervous system diseases: diagnosis by pattern recognition in daily practice

**DOI:** 10.1007/s11604-021-01150-4

**Published:** 2021-06-14

**Authors:** Mio Sakai, Masahiro Higashi, Takuya Fujiwara, Tomoko Uehira, Takuma Shirasaka, Katsuyuki Nakanishi, Nobuo Kashiwagi, Hisashi Tanaka, Hitoshi Terada, Noriyuki Tomiyama

**Affiliations:** 1 Department of Diagnostic and Interventional Radiology, Osaka International Cancer Institute, 3-1-69, Otemae, Chuo-ku, Osaka-shi, Osaka, 541-8567 Japan; 2 Department of Radiology, National Hospital Organization Osaka National Hospital, 2-1-14, Hoenzaka, Chuo-ku, Osaka-shi, Osaka, 540-0006 Japan; 3 AIDS Medical Center, National Hospital Organization Osaka National Hospital, 2-1-14, Hoenzaka, Chuo-ku, Osaka-shi, Osaka, 540-0006 Japan; 4 Department of Future Diagnostic Radiology, Osaka University Graduate School of Medicine, 2-2, Yamadaoka, Suita, Osaka, 565-0871 Japan; 5 Department of Radiology, Osaka University Graduate School of Medicine, 2‑2, Yamadaoka, Suita, Osaka, 565‑0871 Japan; 6 Department of Radiology, Toho University Sakura Medical Center, 564-1, Shimoshizu, Sakura, Chiba, 285-8741 Japan

**Keywords:** HIV, HIV-related central nervous system diseases, AIDS, MRI, Antiretroviral therapy

## Abstract

With the advent of antiretroviral therapy (ART), the prognosis of people infected with human immunodeficiency virus (HIV) has improved, and the frequency of HIV-related central nervous system (CNS) diseases has decreased. Nevertheless, mortality from HIV-related CNS diseases, including those associated with ART (e.g., immune reconstitution inflammatory syndrome) remains significant. Magnetic resonance imaging (MRI) can improve the outlook for people with HIV through early diagnosis and prompt treatment. For example, HIV encephalopathy shows a diffuse bilateral pattern, whereas progressive multifocal leukoencephalopathy, HIV-related primary CNS lymphoma, and CNS toxoplasmosis show focal patterns on MRI. Among the other diseases caused by opportunistic infections, CNS cryptococcosis and CNS tuberculosis have extremely poor prognoses unless diagnosed early. Immune reconstitution inflammatory syndrome shows distinct MRI findings from the offending opportunistic infections. Although distinguishing between HIV-related CNS diseases based on imaging alone is difficult, in this review, we discuss how pattern recognition approaches can contribute to their early differentiation.

## Introduction

In the early 1980, a positive diagnosis of the human immunodeficiency virus (HIV) inevitably led to acquired immunodeficiency syndrome (AIDS) and premature death. HIV causes cellular immunodeficiency primarily by infecting and killing CD4-positive T lymphocytes. The normal absolute serum count for CD4-positive T lymphocytes in adolescents and adults ranges from 500 to 1500 cells/mm^3^. In general, the cell count progressively decreases with the increasing duration of HIV infection. At cell counts < 200 cells/mm^3^, patients are severely immunocompromised and classified as having AIDS [1]. Patients may develop opportunistic infections (OIs) or other HIV-related disorders that can lead to death within a few months without appropriate therapy [1].

Since the introduction of antiretroviral therapy (ART), there has been a significant improvement in the prognosis of HIV-infected patients. ART can reduce or entirely prevent immunosuppression and even improve immune status by suppressing HIV growth [2]. ART transforms HIV infection into a manageable chronic condition, potentially allowing patients to have an almost average life expectancy [3]. However, although ART has notably reduced the prevalence of HIV-related central nervous system (CNS) diseases, secondary to OIs [4, 5], the associated mortality remains high [4]. Therefore, HIV-related CNS diseases are a major cause of death in patients with HIV [6].

Furthermore, nearly 20% of HIV-infected people worldwide are reportedly not diagnosed and treated with ART [7]. Thus, it is not unusual for incidental magnetic resonance imaging (MRI) findings to lead to the suspicion of HIV-related CNS diseases and the subsequent diagnosis of HIV infection [9]. In addition, HIV-infected individuals, who do not adhere to treatment may again become immunodeficient after a few months [8], thus increasing the risk of HIV-related CNS diseases.

MRI, which is minimally invasive and highly reproducible, is one of the most powerful tools for diagnosing and monitoring HIV-related CNS diseases. This article, reviews MRI as a diagnostic tool, focusing on pattern recognition approaches to diagnose HIV-related CNS diseases.

## Clinical and laboratory findings of HIV-related CNS diseases

Information of use in estimating the risk of HIV-related CNS diseases is the serum CD4-positive T lymphocyte count. In patients with counts of < 200 cells/mm^3^, there is a higher risk of HIV encephalopathy, OIs, and primary CNS lymphoma (PCNSL) (Table 1) [10]. Antibody assays of blood serum or cerebrospinal fluid (CSF), polymerase chain reaction assays of CSF, and CSF or blood cultures are recommended for diagnosing some CNS diseases. Some cases may require a biopsy for confirmation and/or empirical treatment [11, 12].

**Table 1:** Correlation between serum CD4-positive T lymphocyte counts and risk of HIV-related CNS diseases [1, 10]

CD4 count (cell/mm^3^)	Frequently occurring CNS diseases
> 500	Same as in immunocompetent hosts
200–500	HIV-associated neurocognitive disorders (HAND)
< 200	Toxoplasmosis, HIV encephalopathy, Cryptococcosis, PML, PCNSL

*HIV* human immunodeficiency virus, *PCNSL* primary central nervous system lymphoma, *PML* progressive multifocal leukoencephalopathy

## Classification with MRI pattern recognition

HIV-related CNS diseases can be classified into three groups based on their etiology (1) impairment directly caused by HIV-related neurologic diseases HIV encephalopathy and HIV vasculopathy, (2) OIs, secondary to HIV CNS toxoplasmosis (CNS-Toxo), progressive multifocal leukoencephalopathy (PML), CNS cryptococcosis (CNS-Crypt), CNS tuberculosis (CNS-TB), PCNSL, and (3) ART-related conditions: immune reconstitution inflammatory syndrome (IRIS) and brain damage caused by ART [1, 13, 14]. Furthermore, they can be classified according to the patterns observed on MRI (Table 2) (1) diffuse and bilateral, (2) focal, and (3) meningitis/meningoencephalitis. HIV encephalopathy shows a diffuse bilateral pattern, whereas PML, HIV-related PCNSL, and CNS-Toxo show focal patterns. However, sometimes, a single disease may have multiple imaging features. For example, CNS-TB and CNS-Crypt, which show meningitis/meningoencephalitis, can present as focal lesions and meningitis. In addition, in some cases, no abnormality is detected on imaging, but such an absence might be an important finding in making a diagnosis. Here, we review MRI findings of HIV-related CNS diseases employing pattern recognition, along with a few exceptions mentioned earlier.

**Table 2:** The most common HIV-related CNS diseases classified by the MRI patterns

MRI pattern	CNS disease
Diffuse and bilateral	HIV encephalopathy
Focal brain lesions	CNS Toxoplasmosis (CNS-Toxo)
	Primary central nervous system lymphoma (PCNSL)
	Progressive multifocal leukoencephalopathy (PML)
Meningitis/meningoencephalitis	CNS-Cryptococcosis (CNS-Crypt)
	CNS-Tuberculosis (CNS-TB)
Others	Immune reconstitution inflammatory syndrome (IRIS)
	HIV-related cerebral infarction

*CNS* central nervous system, *HIV* human immunodeficiency virus, *MRI* magnetic resonance imaging

## Diffuse and bilateral lesions

### HIV encephalopathy

#### Epidemiology and clinical manifestations

HIV encephalopathy is a neurocognitive disorder primarily caused by HIV. HIV enters the CNS within the first few weeks post-infection and causes chronic inflammation [15]. Even on starting ART, many anti-HIV drugs do not cross the blood–brain barrier, and HIV in the brain continues to replicate [2, 3, 16]. In early stages of infection, patients manifest no cognitive symptoms. In later stages, when patients have severe immunodeficiency and manifest cognitive symptoms, pathological findings show HIV leukoencephalopathy characterized by diffuse myelin and axonal degeneration [4]. Since, in many cases, the symptoms improve with ART [3, 4], it is essential to recognize and diagnose the disease at the earliest.

Approximately half of the HIV-infected individuals develop cognitive impairment [15]. Although HIV encephalopathy remains a major cause of cognitive impairment, several comorbidities, such as alcohol and substance abuse; nutritional and vitamin deficiencies, ischemic changes caused by accelerated atherosclerosis due to HIV and ART, and psychiatric illnesses, may also contribute to its development [3]. OIs and PCNSLs may also affect cognition [3]. It has been suggested that chronic inflammation caused by HIV may accelerate age-related changes, and ART may contribute to the deposition of amyloid-β, a hallmark of Alzheimer’s disease, although this theory remains controversial [17]. HIV-associated neurocognitive disorders (HANDs), which correspond to HIV encephalopathy, is a term that has been proposed for medium to long-term cognitive impairment in HIV-infected individuals [18]. It is seen in patients with serum CD4-positive T lymphocyte count between 200 and 500 /mm^3^ [1, 10, 12] (Table 1). The prevalence of HANDs among HIV-infected individuals has been reported to be around 25% [19–23]; the common risk factors are low serum CD4-positive T lymphocytes at the baseline, age of ≥ 50 years, transient increase in viral load, or virological failure [17, 20, 21, 24, 25].

#### Magnetic resonance findings (Fig. 1)

**Fig. 1:**
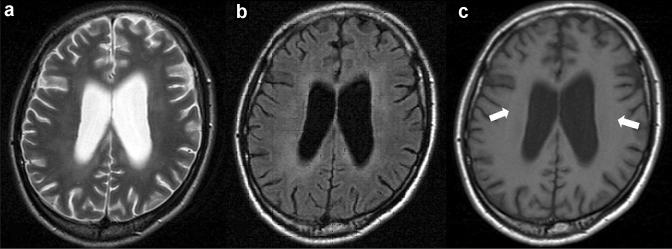
HIV encephalopathy in a 35-year-old man with HIV infection and memory loss. Brain MRI was performed a few days before the initiation of antiretroviral therapy and showed cerebral atrophy. T2-weighted (**a**) and FLAIR images (**b**) showed a pale hyperintense area in the periventricular white matter. The lesion shows an iso to slightly low signal intensity on T1-weighted image (**c**, white arrows)

The diagnosis and evaluation of HIV encephalopathy commonly require neuropsychological testing but not MRI [18, 20, 26]. However, MRI is essential to rule out other causes of cognitive impairment. Conventional MRI often shows no abnormalities, such as mass effects or enhancement, especially in the early stages of the disease. Therefore, if either of these findings is present, other diagnoses should be considered. HIV encephalopathy may be visible on T2-weighted and fluid-attenuated inversion recovery (FLAIR) images, where an increased signal may be seen in the bilateral deep white matter, and rarely, within the subcortical structures or the brain stem (Fig. 1a, b). On T1-weighted images, the lesion shows an iso to slightly low signal (Fig. 1c), this finding is usually apparent in the advanced stages of the disease and is often followed by progressive central-dominant brain atrophy (Fig. 1) [27].

The signal changes in HIV encephalopathy may improve with ART, which may differentiate it from age-related and ischemic changes, however, conventional MRI is not recommended for assessing treatment effectiveness as the symptoms of HIV encephalopathy are not consistently depicted on conventional MRI.

Instead, advanced neuroimaging modalities, such as MR spectroscopy (MRS) [17], diffusion tensor imaging [17, 28], and arterial spin labeling [29] reportedly can be used for evaluating HIV encephalopathy, particularly for monitoring early changes and therapeutic response.

## Focal brain lesions

Among HIV-related CNS diseases, the focal lesions are most commonly found in patients with CNS-Toxo, PCNSL, and PML [30]. PML is a demyelinating disease caused by human polyomavirus 2 (commonly known as the JC virus) infection, and imaging reveals no mass lesions or enhancement. CNS-Toxo and PCNSL may form similar ring-enhanced mass lesions, making the two disorders difficult to differentiate. Since the prognoses of these entities are poor, and they are fatal if left untreated, timely diagnosis is crucial, and appropriate treatment can improve long-term survival [31].

Using other technologies as adjuncts to MRI, such as thallium-201 single-photon emission computed tomography (SPECT) and fluorodeoxyglucose positron emission tomography (FDG-PET), may improve diagnostic accuracy [14, 27, 31–33] (Table 3). The detection of Epstein–Barr virus DNA in the CSF [26, 34] and toxoplasmosis serology can help differentiate CNS-Toxo from PCNSL [26]. However, histopathologic confirmation might still be needed for a definitive diagnosis of PCNSL [14, 31, 34].

**Table 3:** Radiological patterns of HIV-related CNS diseases with focal brain lesions [14, 27, 31–33, 37, 45, 54, 61]

Imaging technique	Toxoplasmosis	PCNSL	PML
MRI findings			
CE	Yes	Yes	No
CE pattern	Ring	Homogenous or ring	No
Edema	Yes	Yes	No
Size	< 4 cm	> 4 cm	N/A
Location	Basal ganglia, thalamus, subcortical WM, cerebellum	Corpus callosum, periventricular WM	Mainly subcortical WM, cerebellar peduncle
Number of lesions	Multiple (occasionally solitary)	Multiple or Solitary	Multiple (occasionally solitary)
SPECT thallium-201	Cold	Hot	Cold
FDG-PET	Hypometabolic	Hypermetabolic	Hypometabolic

*CE* contrast enhancement, *FDG* fluorodeoxyglucose, *HIV* human immunodeficiency virus, *MRI* magnetic resonance imaging, *PCNSL* primary CNS lymphoma, *PET* positron emission tomography, *PML* progressive multifocal leukoencephalopathy, *SPECT* single-photon emission computed tomography, *WM* white matter

### CNS toxoplasmosis

#### Epidemiology and clinical manifestations

*Toxoplasma gondii* is an intracellular parasite that has traditionally been the most common etiological agent of focal CNS disease in patients with AIDS [35]. The relative incidence of CNS-Toxo decreased with improvements in treatment, from 72% in 1991 to 19% in 1996 [14].

The seroprevalence of antibodies against *T. gondii* in the general population varies substantially among different geographic locales, with a prevalence of approximately 11% in the United States and 50–80% in certain European, Latin American, and African countries [11]. Humans may contract the infection by consuming food or water contaminated with oocysts or by eating undercooked meat (pork and lamb) containing tissue cysts. In patients with AIDS, CNS-Toxo primarily arises due to reactivation of latent infection, and the most common clinical feature is focal encephalitis. Patients may also present with nonfocal manifestations, including nonspecific headaches and psychiatric symptoms [11]. Most patients (> 80%) who develop CNS-Toxo have serum CD4-positive T lymphocyte counts of < 100 cells/mm^3^ [14].

A definitive diagnosis of CNS-Toxo requires a compatible clinical syndrome, identification of one or more mass lesions on computed tomography or MRI, and detection of the parasite in clinical samples [11]. In patients with severe immunosuppression, a negative result of anti *T. gondii* antibodies does not exclude the diagnosis of CNS-Toxo because up to 20% of patients with AIDS may not have detectable antibody titers [27].

In clinical practice, patients seropositive for *T. gondii* and CD4-positive T lymphocyte counts < 100 cells/mm^3^ may receive prophylaxis against CNS-Toxo. One study reported a clinical response to acute therapy with oral clindamycin and pyrimethamine in 90% of the patients with CNS-Toxo within 14 days [11, 36].

#### MRI findings (Fig. 2)

**Fig. 2:**
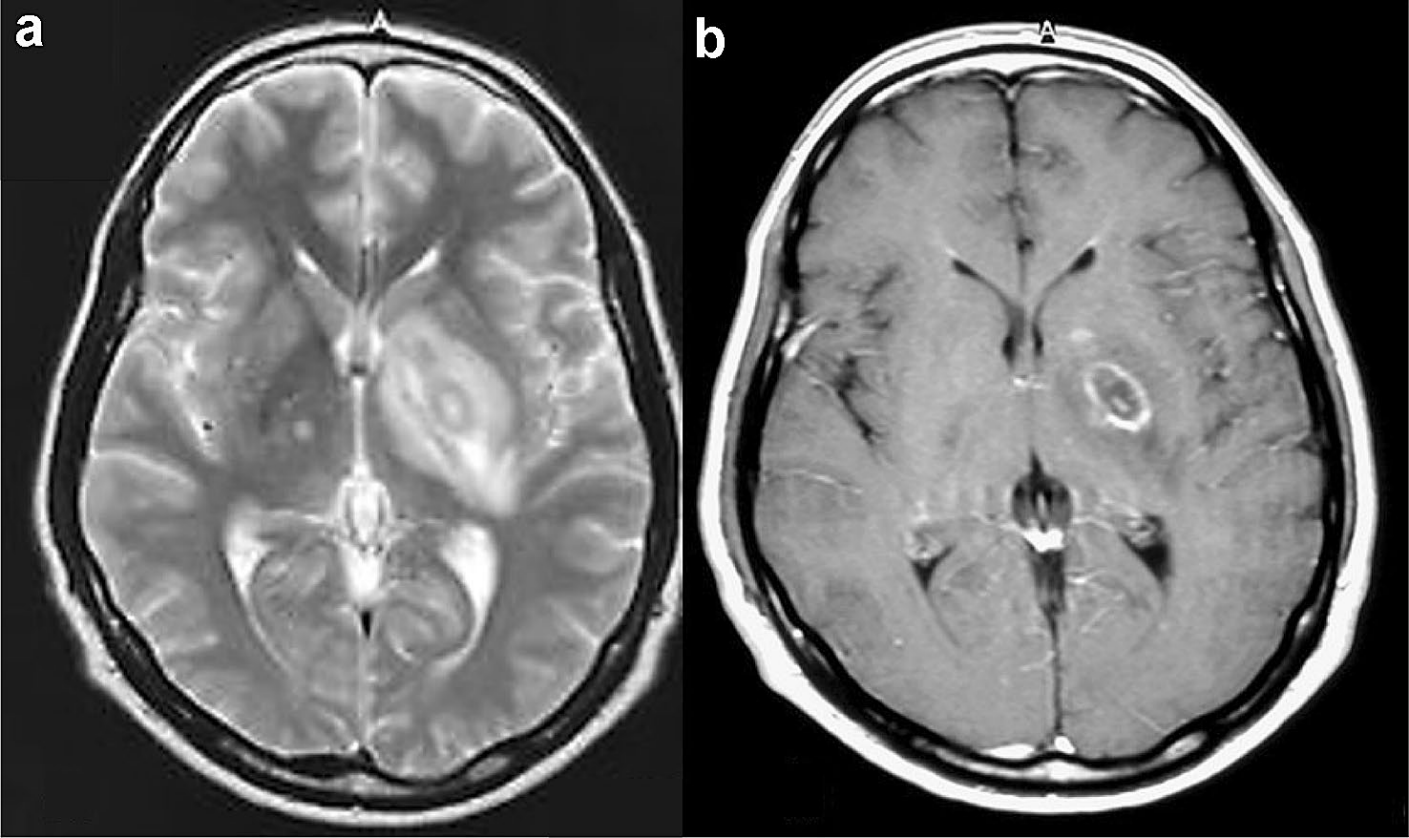
Target sign on T2-weighted image and eccentric target sign on contrast-enhanced T1-weighted image in a patient with HIV-related CNS toxoplasmosis. A mass lesion with surrounding edema was observed in the left lenticular nucleus. On T2-weighted image (**a**), the mass shows a concentric hyper- and hypointense zones with perilesional edema. On contrast-enhanced T1-weighted image (**b**), the mass shows a ring-shaped zone of peripheral contrast enhancement with a small eccentric nodule along the wall. Image b is reprinted from [9] with permission

The characteristic MRI findings of CNS-Toxo are multiple masses, representing *T. gondii* abscesses of 2–3 cm in diameter, with ring contrast enhancement and edema (Table 3), however, single lesions are seen in 15–20% of the cases [27]. The most common sites are the basal ganglia, thalamus, subcortical white matter, and cerebellum [27, 37]. Contrast enhancement may not be seen if the serum CD4-positive T lymphocyte count is < 50 cells/mm^3^. In rare cases, there is extensive encephalitis without abscess formation [35].

The pathognomonic signs for CNS-Toxo are a target sign on T2-weighted images and an eccentric target sign on contrast-enhanced T1-weighted images. The target sign is characterized by concentric high and low signal areas (Fig. 2a), and the number of concentric altered zones varies among reports [38]. The eccentric target sign is characterized by a ring-enhancing lesion with a contrast-enhanced eccentric nodule (Fig. 2b) [39, 40]. The ring corresponds to an inflammatory vascular zone at the edge of a necrotic lesion, and the nodule corresponds to a cluster of thickened vessels [39, 40]. Target sign on T2-weighted images and eccentric target sign on contrast-enhanced T1-weighted image, however, have been observed in only one-third of the cases [31, 39, 40]. Different pathological findings associated with the degree of progression of abscess formation can explain the variety of CNS lesions [39].

### Primary CNS lymphoma

#### Epidemiology and clinical manifestations

PCNSL accounts for up to 15% of non-Hodgkin lymphomas in HIV-infected individuals, and most cases are coinfected with the Epstein–Barr virus [34]. The incidence of PCNSL in HIV-infected individuals is 2–6%, 1,000 times higher than that in the general population [41]. Affected patients typically have serum CD4-positive T lymphocyte counts < 50 cells/mm^3^ [42].

Although ART has decreased the incidence of HIV-related PCNSL, it remains the most common HIV-associated malignancy [42]. Unless treated, the median survival of HIV-infected patients with PCNSL, after the onset of clinical symptoms, is 1 month [41]. With chemotherapy, the median survival increases to 1.5 years [34, 43]. Owing to the rapid progression of the disease, a fast and reliable diagnosis is essential. However, the patients can be asymptomatic or have imperceptible neurologic symptoms [34]. Stereotactic biopsy, CSF testing, cytology, or a combination of these methods are used to diagnose HIV-related PCNSL [34, 44], however, in patients who otherwise have typical clinical and imaging findings, detection of Epstein–Barr virus DNA in the CSF favors the diagnosis of PCNSL.

#### MRI findings (Fig. 3)

**Fig. 3:**
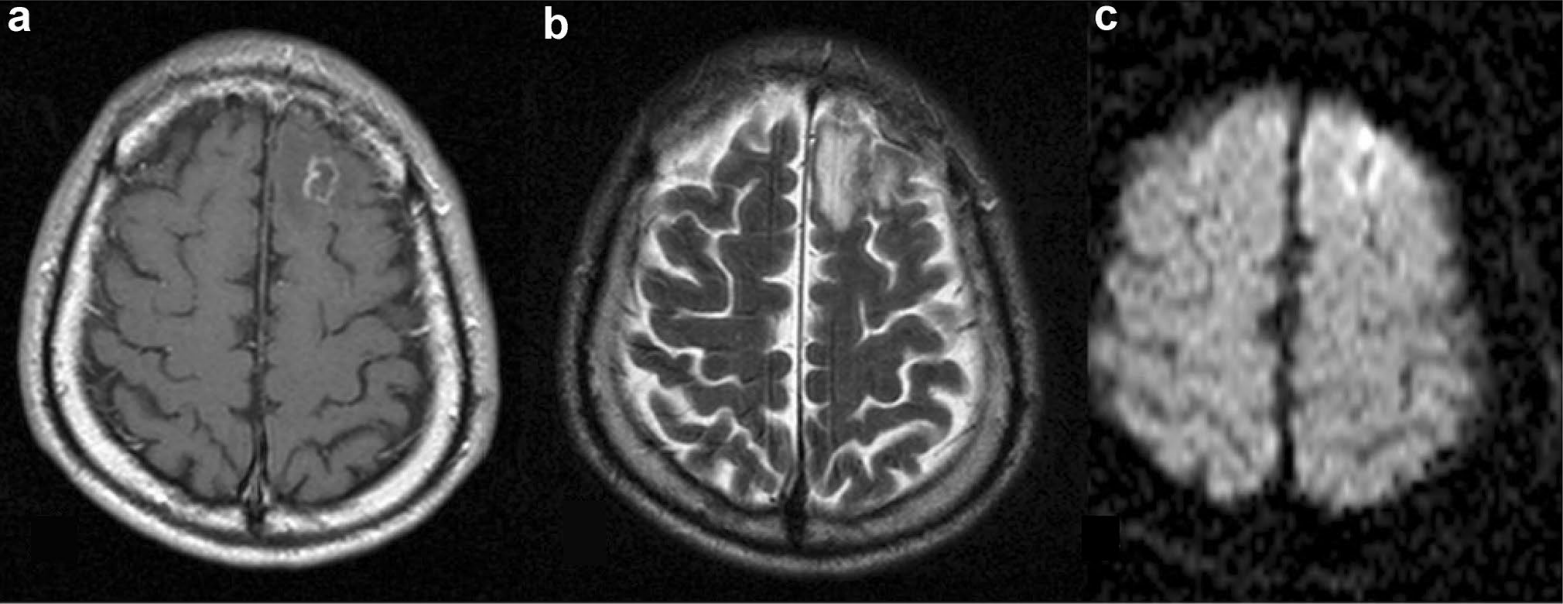
Ring-enhanced lesion in a patient with HIV-related primary CNS lymphoma. Contrast-enhanced T1-weighted image (**a**) shows a ring-enhanced lesion in the left frontal subcortical white matter. The mass shows a heterogeneous signal on T2-weighted image (**b**) with surrounding edema. On diffusion-weighted image (**c**), the lesion shows peripheral hyperintensity

HIV-related PCNSL is characterized by lesions with a larger diameter (≥ 4 cm) than that of CNS-Toxo, with an almost equal likelihood of multiple and solitary lesions [45], they most commonly involve the basal ganglia and corpus callosum [46].

PCNSLs exhibit variable imaging findings based on the patients’ immune status [30]. HIV-related PCNSL is more aggressive than that in immunocompetent patients, and more frequently exhibits central necrosis and spontaneous hemorrhage [30]. When the lesions exhibit necrosis and/or hemorrhage, contrast enhancement is often irregular or peripheral, forming a ring-like enhancement similar to that seen in CNS-Toxo (Fig. 3). It is reported in up to 75% of cases [46], and therefore, differentiating HIV-related PCNSL from CNS-Toxo on conventional MRI can be a diagnostic challenge.

Some additional MR techniques, such as diffusion-weighted imaging (DWI) [47], MRS [48], and perfusion MR imaging [49, 50] are reportedly useful in differentiating HIV-related PCNSL from CNS-Toxo, with a lower apparent diffusion coefficient (ADC) value [47], higher choline/creatinine ratio [48], and higher regional cerebral blood volume (rCBV) in patients with HIV-related PCNSL [50].

However, these findings should be interpreted with caution; particularly, regarding tissue heterogeneity within the lesion caused by necrosis and hemorrhage [49–52]. There is a significant overlap, even in reports wherein DWI-derived ADC values proved statistically useful for differentiation [47, 51]. The authors speculated that necrotic lesions in HIV-related PCNSL are less likely to show restricted diffusion owing to their relative hypocellularity caused by necrosis [51]. When a HIV-related PCNSL lesion is necrotic, portions of the lesion that remain highly cellular may show restricted diffusion, whereas the necrotic portions of the lesion do not [51]**.** Overlapping MRS findings between HIV-related PCNSL and CNS-Toxo have also been reported [52]. The authors speculated that the primary reason for the overlap is that central necrosis in HIV-related PCNSL lesions has a composition similar to that of a necrotic abscess in CNS-Toxo [52]. Tissue inhomogeneity within lesions tends to diminish the specificity of the spectrum [52]. In perfusion MRI, the maximum rCBV is more significant compared with the mean rCBV, which is more susceptible to internal heterogeneity [50].

On thallium-201 SPECT, HIV-related PCNSL shows higher thallium-201 uptake than that of CNS-Toxo [14]. In addition, FDG uptake in HIV-related PCNS is higher than that in CNS-Toxo [31]. However, the accuracy of thallium-201 SPECT and FDG-PET can be affected due to several factors, including the size and location of the lesion and the presence of necrotic and hemorrhagic areas within the tumor [14, 31].

Although these advanced MR techniques and nuclear medicine examinations can aid in diagnosis, a stereotactic biopsy should be performed when the diagnosis is ambiguous, despite with clinical and laboratory findings [53].

### Progressive multifocal leukoencephalopathy

#### Epidemiology and clinical manifestations

PML is a subacute progressive demyelinating disease caused by oligodendrocyte damage resulting from infection with JC virus [54], which has a worldwide distribution and seroprevalence of 39–69% among adults in the general population. Primary JC virus infection usually occurs asymptomatically in childhood, resulting in a chronic asymptomatic carrier state [55], however, it can reactivate in a state of severe immunosuppression. In this setting, neurotropic variants that can replicate in the glial cells may form [56]. Then, the virus spreads to the brain and induces a lytic infection of oligodendrocytes, which are myelin-producing cells [56].

HIV infection is strongly associated with PML; during the AIDS pandemic, it developed in 5% of HIV-infected individuals. This association might, in part, be explained by the synergistic effect of HIV and JC virus co-infection. Outside the context of HIV infection, PML characteristically manifests as a complication of other immunocompromising conditions. In the past 4 decades in Western countries, HIV infection accounted for approximately 80% of PML cases [57], whereas in Japan, HIV infection accounted for one-third of PML cases [58]. Based on postmortem data, the prevalence of PML in patients with AIDS was between 2.4 and 5.3% [14].

No specific therapy exists for JC virus infection or HIV-related PML. The primary therapeutic approach involves ART to reverse immunosuppression that interferes with the normal host response to JC virus [55]. Unless treated, HIV-related PML results in death within approximately 2.5–4.0 months of disease onset [14]. With ART, survival in patients with HIV-related PML has extended to years [54, 55, 57]. Among survivors of HIV-related PML, 44%–83% achieve clinical stabilization or some improvement if ART was initiated early in the disease course. However, many long-term survivors are left with substantial morbidity—approximately 70% experience some residual neurological disability, which is moderate to severe in 25–50% of cases. Up to 44% of survivors experience seizures [57]. Thus, early diagnosis and prompt initiation of ART are key factors for preserving neurological function in patients with PML [54].

PML has an insidious onset and steady subacute progression and manifests as focal neurological deficits. Since demyelinating lesions can involve any region in the brain, neurological disabilities vary from patient to patient. However, a common finding is that the symptoms worsen daily due to the progression and spread of demyelinating lesions [55].

#### MRI findings (Figs. 4, 5)

**Fig. 4:**
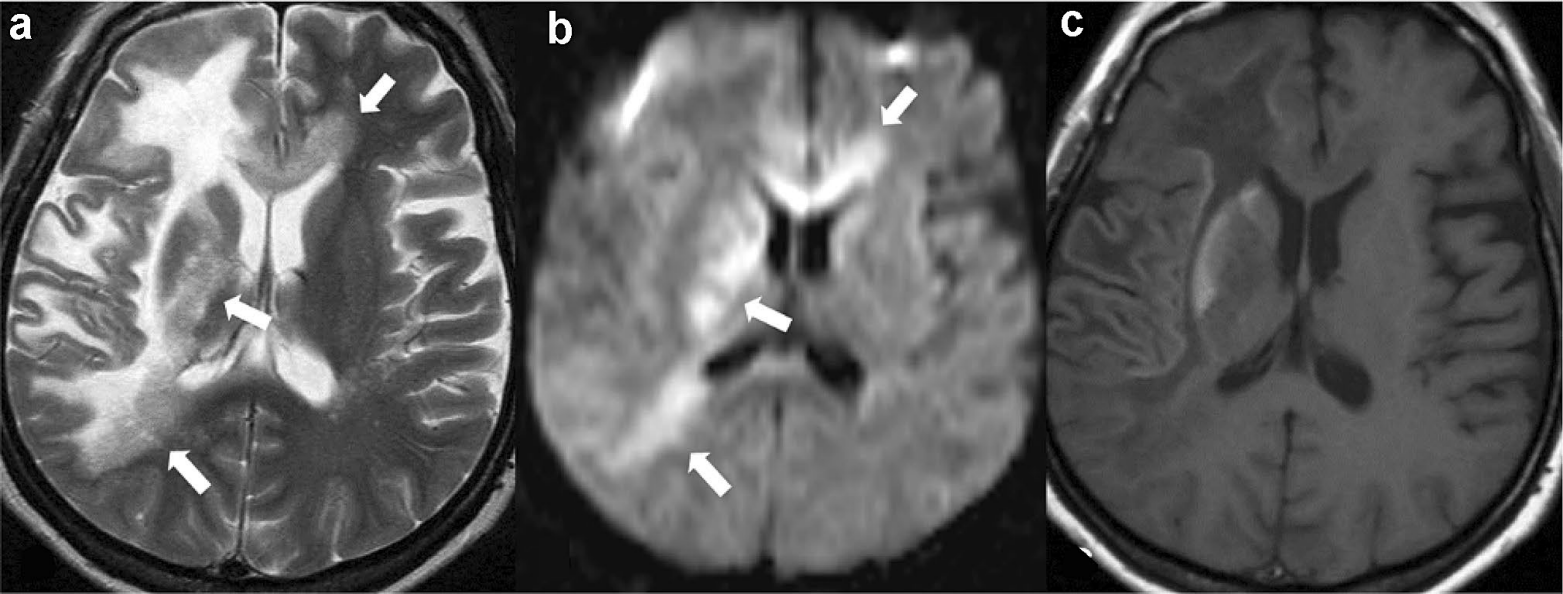
Heterogeneous demyelination in a patient with HIV-related progressive multifocal leukoencephalopathy. On T2-weighted image (**a**), the older demyelinated lesion shows homogenous hyperintensity, while its medial margin, frontline (white arrows), shows heterogeneous pale hyperintensity with numerous punctate hyperintensities (milky way appearance). On diffusion-weighted image (**b**), the hyperintensity area (DW hyperintensity rim) corresponding to the milky way appearing area is shown. On T1-weighted image (**c**), hyperintensity is seen in the right head of the caudate nucleus, putamen, and subcortical area. They are adjusted to a large confluent demyelinated area showing homogenous hyperintensity on T2-weighted image (**a**) and hypointensity on T1-weighted image (**c**). The image a and b were reprinted from [9] with permission

**Fig. 5:**
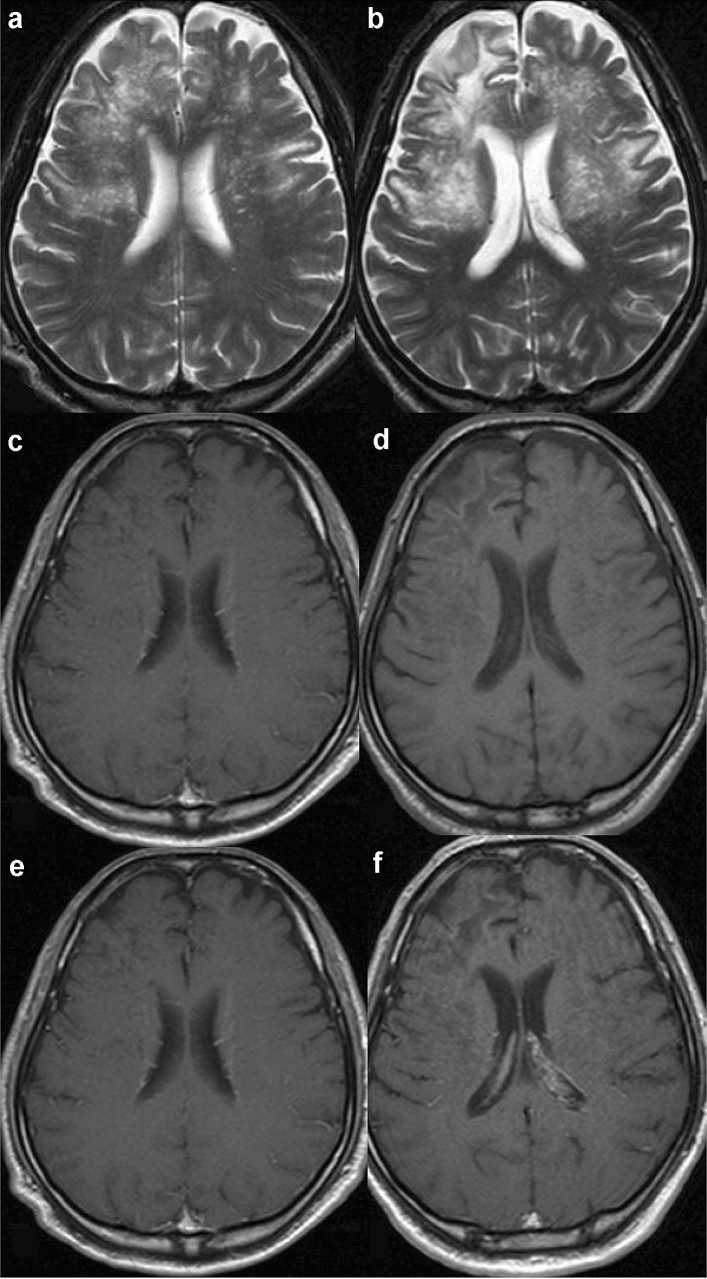
Lesion progression in a patient with HIV-related progressive multifocal leukoencephalopathy. T2-weighted (**a**, **b**), T1-weighted (**c**, **d**), and contrast-enhanced T1-weighted (**e**, **f**) images acquired 2 months (**a**, **c**, **e**) and 3 months (**b**, **d**, **f**) after the initial symptom of facial palsy. The first T2-weighted image (**a**) shows numerous dots with pale hyperintense lesions (milky way appearance) involving the bilateral frontal white matter predominantly on the right. The first T1-weighted image (**c**) shows hypointense lesions corresponding to advanced demyelination in right subcortical white matter. The second images (**b**, **d**, **f**) show more expansive lesions with more advanced demyelination than the first images (**a**, **c**, **e**). Milky way appearance is more prominent in the left frontal white matter on the second T2-weighted image (**b**) than on the first T2-weighted image (**a**)

MRI is a powerful tool for the diagnosis of PML. Diagnostic criteria, based on clinical, MRI, and CSF findings, have been broadly adopted, in addition to classical histopathological criteria [57, 59].

MRI appears as distinct white matter lesions in areas of the brain that correspond to neurological symptoms. Without therapy, the lesions expand subacutely and are accompanied by the worsening of clinical symptoms [54, 57]. The diagnostic classification includes several levels of certainty (Table 4) [59]. When typical clinical and MRI findings are present, the diagnosis of PML can be based on the presence of JC virus DNA in the CSF [57].

**Table 4:** Summary of the diagnostic criteria for PML [57, 59]

Certainty of PML diagnosis	Compatible clinical features	Compatible MRI findings	CSF PCR for JC virus	Alternative diagnoses are clinically excluded
Definite	+	+	+	+
Probable	+	−	+	+
	−	+	+	+
Possible	+	+	−/ND	+
	−	−	+	+
Not PML	−	−	−	−
	+	−	−	−
	−	+	−	−

+ indicates present, −indicates absence

*CSF* cerebrospinal fluid, *JCV* JC virus, *ND* not done or equivocal result, *PCR* polymerase chain reaction, PML progressive multifocal leukoencephalopathy

The characteristic MRI finding for PML is the presence of asymmetrically located white matter lesions. The lesions show a low signal on T1-weighted image and a high signal on T2-weighted images [59]. Contrast enhancement is generally negative, but faint enhancement may be seen at lesion margins [60]. It may be challenging to differentiate PML from other diseases, such as gliomatosis or multiple sclerosis with imaging findings alone. However, subacute lesion progression on repeat MRI accompanied by worsening clinical symptoms and the uniquely uneven degree of demyelination within the lesion is often shown in PML [54, 57].

Although demyelination by JC virus can occur anywhere oligodendrocytes exist, the lesions often first appear in the subcortical white matter of the cerebrum, including in the short-range association fibers (also called U-fibers) [54]. The lesions are often distributed asymmetrically, and even when the distribution is bilateral and symmetrical, the degree of demyelination is asymmetrical (Fig. 4) [54]. Demyelination spreads outwards, from the lesion to the surrounding area.

The newer, spreading demyelinating margin is called the frontline, whereas older portions of the lesions show advanced demyelination (Fig. 4a, b) [54]. The frontline margins on T2-weighted images show a diffuse pale hyperintensity and/or numerous discrete hyperintense dots giving a milky way appearance [54]. This often corresponds to the hyperintense rim on DWI (Fig. 4a, b) [27, 61]. In many cases, the hyperintense rim on DWI is interrupted at older demyelinated margins. With progression, the intensity gradually increases on T2-weighted images and coalesces to form a homogeneous advanced demyelinated lesion of near-CSF intensity (Fig. 5) [65].

The cerebral cortex and deep-gray matter bordering advanced demyelinated areas may show a high signal on T1-weighted images (Fig. 4c) [54, 62]. Hypointense signals may also be visualized on susceptibility-weighted imaging for deep layers of the cerebral cortex bordering advanced demyelinated areas [63, 64]. These findings on T1-weighted and susceptibility-weighted images are not specific to PML or directly due to JC virus infection, but they may be secondary to demyelination [64].

## Meningitis/meningoencephalitis

A wide range of pathogens can cause meningitis/meningoencephalitis in HIV-infected patients, but here, we will discuss CNS-Crypt and CNS-TB, because of their impact on acute mortality. Although the overall prognosis of HIV-related CNS-Crypt and CNS-TB has improved since the introduction of ART, acute mortality has not changed significantly over time, ranging 6–16% for CNS-Crypt [66–70] and at 30% for CNS-TB [71].

If HIV-infected patients have unexplained fever, especially those with severe immunosuppression, these disorders should be actively suspected and investigated immediately [71, 72]. Both CNS-Crypt and CNS-TB are diagnosed by pathogen detection in the CSF. When meningitis is suspected in HIV-infected patients, CSF should be tested for antibodies against pathogens causing both diseases [73]. Since both CNS-Crypt and CNS-TB usually disseminate from lung lesions, chest computed tomography and radiography may trigger the diagnosis [27, 72, 74–77]. Combining CSF pathogen testing with chest imaging may confirm the suspicion and allow early treatment initiation [74, 77].

### CNS-Crypt

#### Epidemiology and clinical manifestations

HIV infection is the most common risk factor for cryptococcosis caused by *Cryptococcus neoformans*. Although *Cryptococcus gattii* is the other primary cause of cryptococcosis in humans, it is mainly reported in immunocompetent individuals exposed to plant propagules found in tropical and subtropical regions [77–81]. *Cryptococcus* spp. enters the body by inhalation, and in most cases, is eliminated by host defense mechanisms. However, in some cases, especially in HIV-infected immunocompromised individuals, it may lead to pneumonia and subsequent CNS dissemination, causing meningoencephalitis. Although HIV infection is the major risk factor for CNS-Crypt, spread to the CNS is also observed in immunocompromised HIV-negative and immunocompetent individuals [77].

HIV-related CNS-Crypt occurs when the serum CD4-positive T lymphocyte cell count drops below 50–100 cells/mm^3^ [82]. HIV-related CNS-Crypt is characterized by a low-grade inflammatory response [83]. Therefore, symptoms other than fever are often minimal and meningococcal irritation is often absent [72, 83].

#### MRI findings (Fig. 6)

**Fig. 6:**
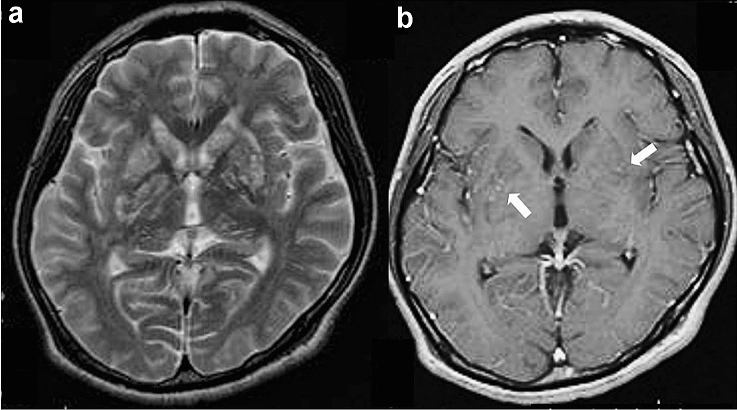
Dilated periventricular space in a patient with HIV-related CNS Cryptococcosis. T2-weighted image (**a**) shows clustered innumerable hyperintense cysts, characteristic of cryptococcal gelatinous pseudocysts, bilaterally in the lentiform nuclei and head of the caudate nucleus and thalamus. Some of them show contrast enhancement on contrast-enhanced T1-weighted image (**b**, arrows)

The radiologic manifestations of CNS-Crypt vary and are frequently minimal [27, 84]. MRI findings are nonspecific or, most often, normal [85]. In HIV-infected patients with fever, CNS-Crypt should be considered irrespective of positive or negative MRI findings [43].

The positive imaging findings in CNS-Crypt correspond to three main forms meningitis/meningoencephalitis, gelatinous pseudocysts, and a granuloma called cryptococcoma. Among them, meningitis/meningoencephalitis and gelatinous pseudocysts are seen in HIV-related CNS-Crypt. Mass lesions called cryptococcoma due to *C. neoformans* are rarely seen in patients who are not receiving ART [27, 86].

Meningitis/meningoencephalitis is the primary lesion of CNS-Crypt and is pronounced at basal cisterns. If the meningeal infection spreads along the perivascular spaces, gelatinous mucoid-like cryptococcal capsular polysaccharides and budding yeast accumulate within dilated perivascular spaces and gives rise to small cysts called gelatinous pseudocysts. These cysts exhibit a “soap bubble appearance” on MRI, with a low to intermediate signal on T1-weighted images, a high signal on T2-weighted images, and a low signal on FLAIR images [87]. Gelatinous pseudocysts are often found in the basal ganglia, thalamus, and midbrain. In patients with severe immunosuppression, the only positive imaging findings indicating CNS-Crypt are mild/non-enhancing cystic lesions and meninges and bilateral symmetrical perivascular space enlargement (Fig. 6) [43].

When there is some degree of the immune response, such as when ART has already been administered, imaging may show cryptococcoma and nodular meningeal enhancement similar to that seen in granulomatous diseases (e.g., tuberculosis, syphilis, and sarcoidosis) and carcinomatous meningitis [27, 86].

### CNS tuberculosis

#### Epidemiology and clinical manifestations

CNS-TB is caused by *Mycobacterium tuberculosis*, disseminated through the bloodstream from the tuberculosis foci in the lungs. Although the most common manifestations of CNS-TB are meningitis, tuberculoma and abscess are not unusual (occurring in approximately 25 and 20% of the cases, respectively) and can be seen separately or simultaneously [27, 88]. HIV-infected individuals are up to 20 times more likely to fall ill with TB. In 2017, 10 million people developed TB globally, 9% of whom had HIV [89].

#### MRI findings (Figs. 7, 8)

**Fig. 7:**
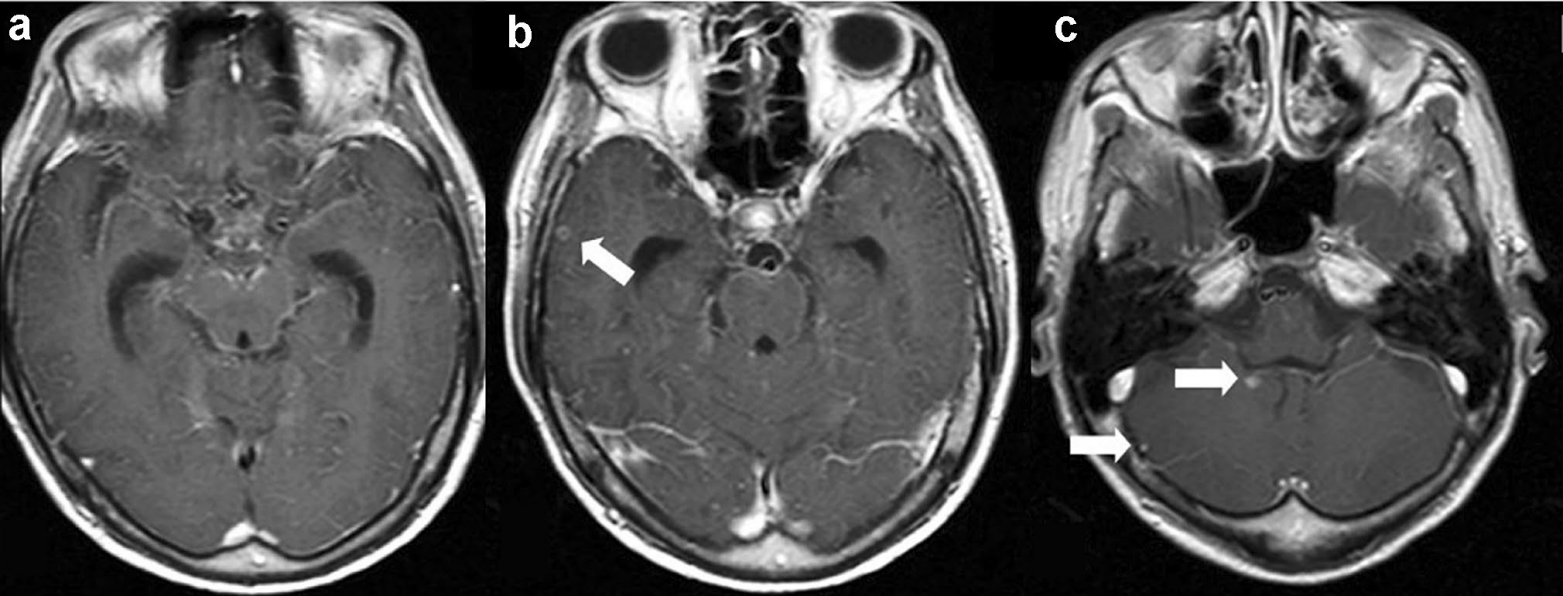
Basilar meningitis and tuberculoma in a patient with HIV-related CNS tuberculosis. Contrast-enhanced T1-weighted image of the basal cistern level (**a**) shows contrast enhancement along the midbrain, and medial temporal lobe representing meningitis. Peripherally located contrast-enhanced nodules representing tuberculomas are also shown (**b**, **c**, arrows)

**Fig. 8:**
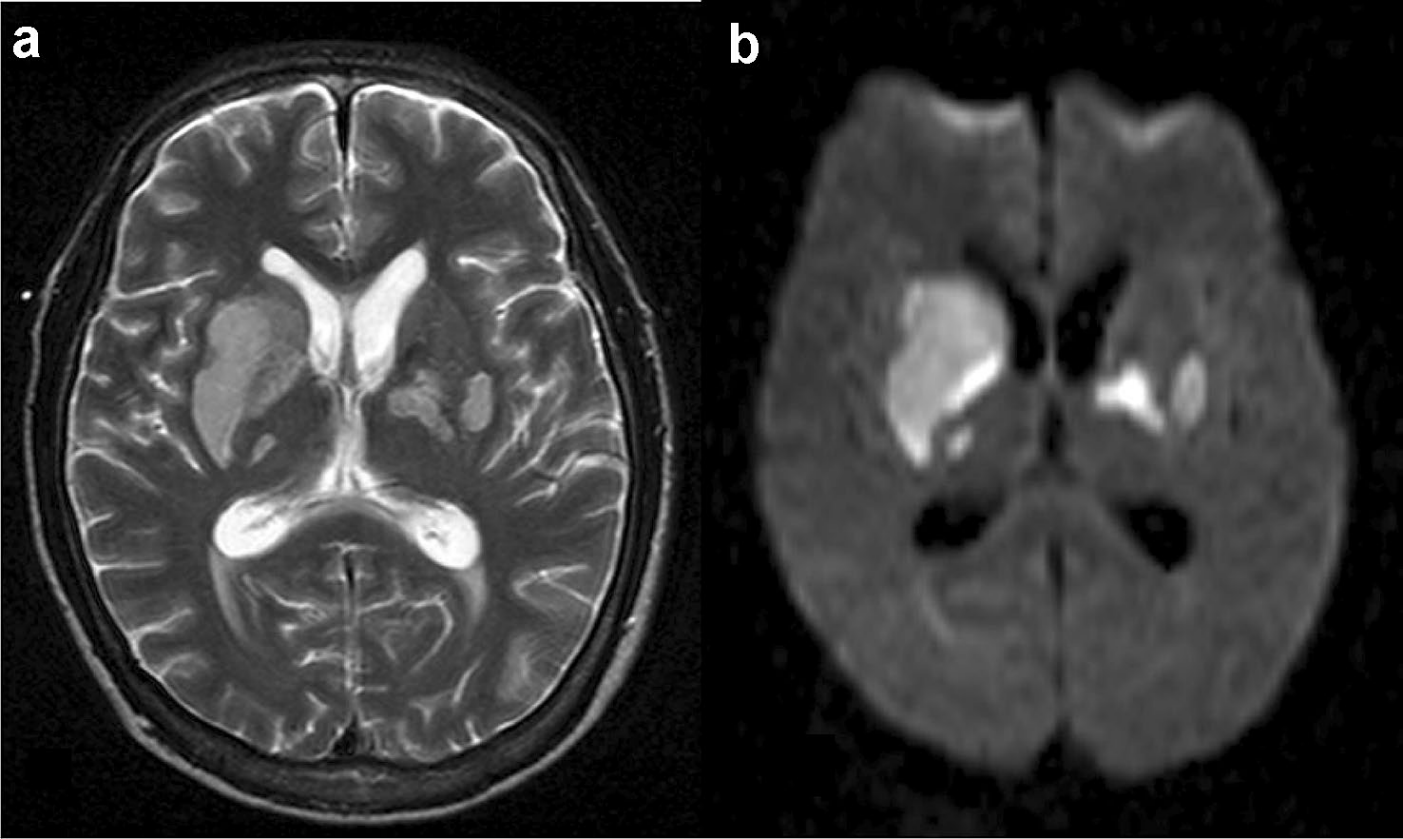
Acute infarction caused by tuberculosis meningitis in a patient with HIV infection. Hyperintensity on T2-weighted image (**a**) is shown in the bilateral basal ganglia with slight swelling. The lesion shows hyperintensity on diffusion-weighted image corresponding to acute infarction (**b**). Enlargement of the bilateral lateral ventricle revealing hydrocephalus was also noted

In patients with CNS-TB with meningitis, classic neuroimaging findings include hydrocephalus (75%), basilar exudates (38%), periventricular infarcts (15–30%), and cerebral parenchymal tuberculomas (5–10%), which might be seen separately or simultaneously [88, 90–92]. Although imaging findings in HIV and non-HIV-associated CNS-TB are almost similar, meningitis is more frequent in those infected with HIV. In a study of 2205 patients with TB, 10% of the HIV-positive patients had TB meningitis, compared with only 2% of the non-infected HIV patients [93]. In contrast to CNS-Crypt, CNS-TB frequently shows hydrocephalus (similar to non-HIV-related CNS-TB) [43] and meningeal contrast enhancement, especially in the basal cisterns (Fig. 7a) [27], their presence is strongly suggestive of tuberculous meningitis [91, 92]. On contrast-enhanced T1-weighted images, tuberculomas with caseous necrosis show a ring-shaped contrast enhancement, whereas those without caseous necrosis show homogeneous contrast enhancement (Fig. 7b, c) [27].

Inflammation can also involve blood vessels, and impaired circulation and vasospasm may lead to infarction, which is often bilateral in the basal ganglia (Fig. 8) [94].

## Immune reconstitution inflammatory syndrome

Iyndrome is a major complication related to ART and is a consequence of excessive activation of the immune system against persistent antigens (paradoxical IRIS), viable pathogens (unmasking IRIS), or self-antigens [95, 96]. The prevalence of IRIS ranges from 7.8 to 13% [86–88], and onset occurs 1–1.5 months after ART initiation [96]. Clinical factors associated with IRIS development are a serum CD4-positive T lymphocyte count of < 50 cells/mm^3^ and HIV-RNA levels greater than 1.0 × 10^5^ copies/mL (high viral load) at the time of ART initiation [96].

### CNS-IRIS

#### Epidemiology and Clinical Manifestations

CNS-IRIS has an acute onset and it rapidly progresses from symptom onset to death [97]. The syndrome may be recognized by the development of new or worsening of existing clinical symptoms (e.g., OIs) despite adequate treatment and serological response. Specific abnormalities can be found on MRI or computed tomography images [98]. Glucocorticosteroids may be used in patients with severe IRIS symptoms [99]. Patients should also be monitored for other OIs that may develop during therapy.

Among HIV-related CNS diseases, PML and CNS-Crypt are the most frequently encountered diseases. IRIS develops in at least 18% of HIV-infected patients with PML after starting ART [95]. As ART is the only effective therapy for PML, dealing with IRIS is especially difficult in these patients [95]. In patients with HIV-related CNS-Crypt, IRIS is estimated to occur in 16.7% of patients after ART initiation [99, 100]. Less frequently reported pathogens associated with CNS-IRIS are varicella-zoster virus, cytomegalovirus, *Candida* spp., *Mycobacterium tuberculosis*, and *Toxoplasma gondii* [96, 101]. Although not in the brain, IRIS develops in more than one-third of patients with retinitis caused by cytomegalovirus [96].

#### MRI findings (Fig. 9)

**Fig. 9:**
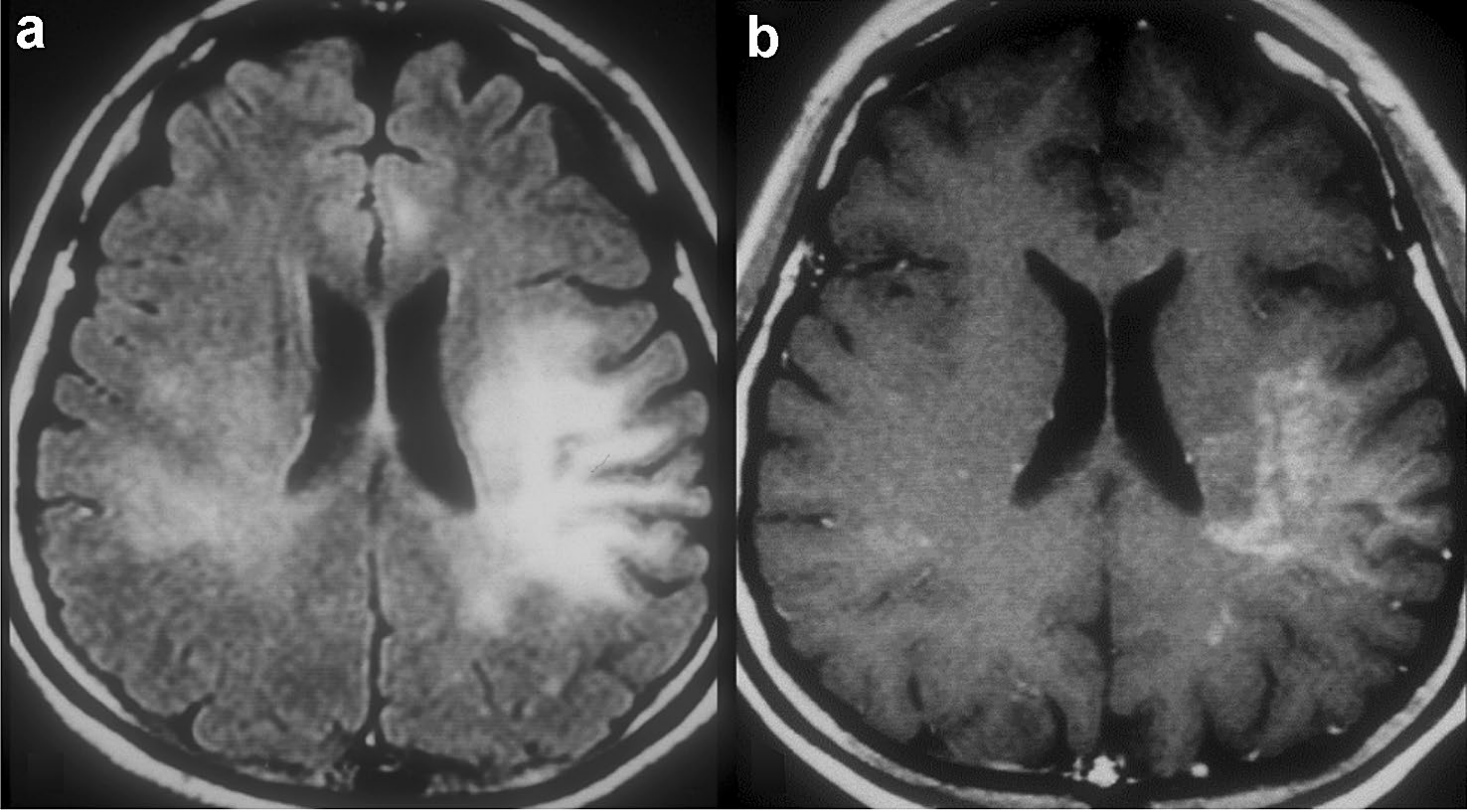
Immune reconstitution inflammatory syndrome (IRIS) developed in a patient with progressive multifocal leukoencephalopathy. FLAIR image (**a**) shows white matter lesions due to PML. Contrast-enhanced T1-weighted image (**b**) shows parenchymal and perivascular enhancement (**b**) compatible with IRIS

On MRI, the presence of an enhanced area on contrast-enhanced T1-weighted images, a transient increase in parenchymal abnormalities with a high signal on T2-weighted or FLAIR images, mass effect, and peripherally restricted diffusion on DWI can be valuable clues to diagnose CNS-IRIS (Fig. 9) [95]. However, a negative MRI finding alone should not exclude the diagnosis [95]. Although CNS-IRIS is a diagnosis of exclusion, MRI can be pivotal for early recognition and improved prognosis [95]. In addition, a contrast-enhancing effect is not always associated with a poor prognosis [102]. In some studies, contrast enhancement was reported to be associated with a good prognosis [103]. These findings might indicate immune recovery, and careful monitoring of the clinical course along with imaging findings is recommended.

## Extra-CNS findings on brain MRI

During brain MRI reading, attention should also be paid to the cranial bone and extracranial lesions, particularly those involving the bone marrow, parotid glands, lymphoid tissue, and skin. HIV causes chronic inflammation and hyperplasia of the bone marrow [104–106], both of which could result in a decreased signal on T1-weighted images, making the bone marrow appear “gray” [107]. In addition, HIV and ART both increase the risk of osteoporosis [108]. The frequency of parotid lesions in HIV-infected patients is reported to be 1–10%, and in some cases distinguishing them from other bilateral parotid tumors, such as Warthin cysts, is difficult [109]. Kaposi’s sarcoma, a vascular neoplasm caused by human herpesvirus type eight, is the most common AIDS-related malignancy and is often found in the head and neck area.

## Conclusions

In summary, HIV encephalopathy shows a diffuse bilateral pattern, whereas PML, PCNSL, and CNS-Toxo show focal patterns on MRI. Among meningitis/meningoencephalitis, that have extremely poor prognosis, CNS-Crypt commonly shows no abnormalities on imaging, while in many cases, CNS-TB shows findings suggestive of meningitis. CNS-IRIS displays distinct MRI findings of the offending OIs. With this knowledge, radiologists may play a pivotal role in the early recognition of HIV-related CNS diseases and thus contribute to a better prognosis for patients.
